# Methylglyoxal Mediates Adipocyte Proliferation by Increasing Phosphorylation of Akt1

**DOI:** 10.1371/journal.pone.0036610

**Published:** 2012-05-14

**Authors:** Xuming Jia, Tuanjie Chang, Thomas W. Wilson, Lingyun Wu

**Affiliations:** 1 Department of Pharmacology, Collage of Medicine, University of Saskatchewan, Saskatoon, Canada; 2 Department of Medicine, Collage of Medicine, University of Saskatchewan, Saskatoon, Canada; 3 Department of Health Sciences, Lakehead University and Thunder Bay Regional Research Institute, Thunder Bay, Canada; University of Pecs Medical School, Hungary

## Abstract

Methylglyoxal (MG) is a highly reactive metabolite physiologically presented in all biological systems. The effects of MG on diabetes and hypertension have been long recognized. In the present study, we investigated the potential role of MG in obesity, one of the most important factors to cause metabolic syndrome. An increased MG accumulation was observed in the adipose tissue of obese Zucker rats. Cell proliferation assay showed that 5–20 µM of MG stimulated the proliferation of 3T3-L1 cells. Further study suggested that accumulated-MG stimulated the phosphorylation of Akt1 and its targets including p21 and p27. The activated Akt1 then increased the activity of CDK2 and accelerated the cell cycle progression of 3T3-L1 cells. The effects of MG were efficiently reversed by advanced glycation end product (AGE) breaker alagebrium and Akt inhibitor SH-6. In summary, our study revealed a previously unrecognized effect of MG in stimulating adipogenesis by up-regulation of Akt signaling pathway and this mechanism might offer a new approach to explain the development of obesity.

## Introduction

Methylglyoxal (MG) is a reactive dicarbonyl compound that interacts with certain free amino acid residues in proteins and forms advanced glycation endproducts (AGEs) [1]. It is derived from glycolysis as well as lipid and protein catabolism [2], [3]. MG-induced reactive oxygen species (ROS) [4], [5], [6], [7] and MG-derived protein modifications [8], [9] have been addressed as possible causal factors for insulin resistance *in vitro* and *in vivo*. Moreover, increased accumulation of MG and AGEs were observed in diabetic [10], [11], [12] and hypertensive [11], [13], [14] animals and patients. The full name of alagebrium is 3-(2-oxo-2-phenyl)- ethyl-4, 5-dimethyl-thiazolium chloride. It is a stable derivative of *N*-phenacylthiazolium bromide (PTB). For its significant effects of reducing AGEs *in vivo* and *in vitro* [15], [16], alagebrium has been applied in animal and clinical studies to treat hypertension and cardiovascular complications by regaining flexibility and functionality of the vascular system (13).

As the most important risk factor for hypertension and diabetes, obesity is well recognized as a result of excessive consumption of fat and carbohydrates, which are both precursors of MG and AGEs. The association between obesity and diabetes and hypertension led us to postulate a possible role of MG in the development of obesity. The development of obesity involves both adipocyte hypertrophy and hyperplasia [17], [18]. While imbalanced energy intake-induced adipocyte hypertrophy is responsible for most adult-onset obesity, obesity in childhood may be due to adipocyte hyperplasia [19], [20]. However, proliferation of adipocytes is also observed in adult obesity. Recently, the roles of PI3K/Akt pathway and its downstream effectors in adipogenesis especially the proliferation of pre-adipocytes were reported [21], [22], [23], [24], [25], [26]. It was found that Akt phosphorylates cyclin-dependent-kinase (Cdk) inhibitors, p27 and p21, prevents the localization of these proteins to nucleus, and attenuates their inhibitory effect on Cdk2. Thus, the cell cycle progression from G_1_ to S phase is accelerated [27], [28], [29]. As Akt1 is one of the main Akt isoforms that related to cell proliferation, it is likely that MG may act through modifying the Akt1 activity and resulted in cell proliferation and growth.

An inhibitory effect of MG on cell growth by inducing apoptosis was studied [30], [31], [32]. It is reported that MG promoted programmed cell death through several signaling pathways including growth factor receptor and the gp130/STAT3-signaling pathway. However, the concentrations of MG (100 to 500 µM) causing apoptosis were much greater than the physiological concentration, which is 1.81–3.29 µM in normal Sprague-Dawley rats [33] and 3.3–5.9 µM in healthy and diabetic humans [12]. The effects of MG at physiological or pathological levels on cell growth need to be further explored. In the present study, we used 3T3-L1 cells, a widely used adipocyte-like cell line, to test whether MG contributes to the development of obesity through stimulating adipocyte proliferation [34].

## Results

### Increased Akt1 Phophorylation Associated with MG Accumulation in Obese Rats

**Figure 1:**
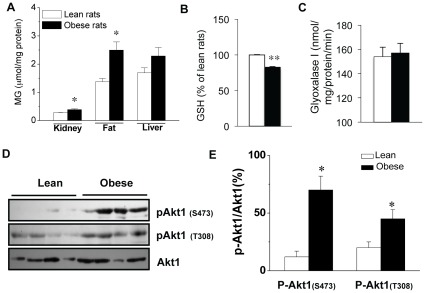
Increased MG accumulation, reduced GSH level and glyoxalase I activity were related to Akt1 expression in obese Zucker rats. (***A***) MG levels in kidney, fat, liver of 16-week old Zucker lean or obese rats. *P<0.05, n = 4−8 in each groups. (***B***) GSH level decreased in the adipose tissue of Zucker obese rats while glyoxalase I activity (***C ***) remain unchanged compare with Zucker lean rats. GSH level was presented as % of that in control group. *P<0.05, n = 4 in each groups. (***D***) The expression of p-Akt1 and Akt1 in adipose tissue of lean and obese Zucker rats. *P<0.05, **P<0.01, n = 4 in both groups. The results of Western blotting were quantified by Chemigenus® Bio imaging system company) and presented as the percentage of that from control cells (***E***). □ Zucker lean rats, ▪ Zucker obese rats.

It was reported that plasma level of MG was increased in rats with diabetes and hypertension [12], [35], [36], [37], [38]. To examine the correlation between MG accumulation and the development of obesity, we compared the MG accumulation in the white fat tissues from Zucker lean and obese rats. At the age of 16 weeks, the body weight of the obese rats was significantly greater than that of the lean rats (Table 1), which is consistent with an increased adipose tissue deposit in obese rats (data not shown). Obese rats also exhibited higher serum triglyceride (TG), higher total cholesterol (Chol), but significantly decreased high-density cholesterol (HDL) level comparing with those of lean rats (Table 1). Although the fasting glucose level did not show significant difference between lean and obese rats at the ages of 10, 12, 14 and 16 weeks (Table 1), a markedly increased MG accumulation was observed in kidney, and fat tissue of obese rats at age of 16 weeks (Fig. 1*A*). In addition, MG level was shown accumulated in serum of obese rats (13.46±1.13 µM *vs* 4.08±0.94 µM in lean rats). With the increased MG accumulation, a decreased GSH level was observed in obese rats (Fig. 1*B*). However, the activity of glyoxalase I, the major enzyme detoxifying MG, did not show significant change (Fig. 1*C*), suggesting that elevated MG level may be mainly related to MG formation.

**Table 1:** Basic parameters of lean/obese Zucker rats.

	Bodyweight (g)	Lipid profile (mM)			Blood glucose (mM)			
		Chol	TG	HDL	10	12	14	16wk
**Lean**	376.4±9.3	1.2±0.0	0.4±0.1	1.0±0.05	5.4±0.1	5.6±0.8	5.0±0.2	5.7±0.2
**Obese**	600.5±5.7*	2.3±0.1*	5.5±1.2**	0.1±0.1**	5.6±0.2	5.5±0.2	5.8±0.5	6.3±0.4

* *P*<0.05,

** *P*<0.01 vs lean Zucker rats, n = 4−8 in each group.

Chol: total cholesterol, TG: triglyceride, HDL: high density lipoprotein.

As Akt1 is the isoform that contributes to cell proliferation and cell growth, we studied the phosphorylation levels of Akt1 isoform in adipose tissues from Zucker lean and obese rats at the age of 16 weeks. Significantly elevated levels of phospho-Akt1(S473) and phospho-Akt1(T308), two activation sites of Akt1 kinase, were observed in Zucker obese rats as compared to that of Zucker lean rats (Fig. 1*D,E*).

### MG Stimulated Proliferation of Cultured 3T3-L1 Cells

**Figure 2:**
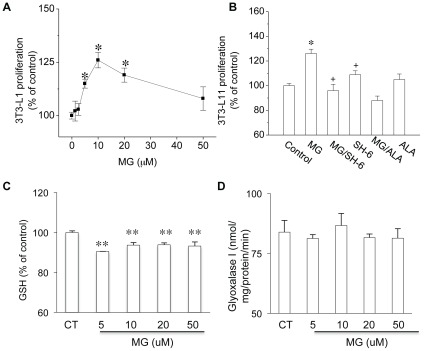
The effect of MG on 3T3-L1 cell proliferation, GSH level and glyoxalase I activity. The relative cell proliferation of each group was presented as the ratio between arbitrary absorbance on 570 nm of each group and that from the control group without treatment. The effect of different MG concentrations on cell proliferation was shown in (***A***) and the effect of 10 µM MG with/without SH-6 and alagebrium was shown in (***B***). The reduced GSH level (***C ***) and unchanged glyoxalase I activity (***D***) was observed in 3T3-L1 cells treated with 5, 10, 20 and 50 µM MG. *P<0.05, **P<0.01 *vs* control cells; **^+^** *P*<0.05 *vs* MG treated cells; n = 12 in each group.

To investigate whether MG treatment could induce the proliferation of 3T3-L1 cells, we carried out a cell proliferation assay with or without MG treatment (1.25∼50 µM). MG at 5, 10 and 20 µM increased the proliferation rate of 3T3-L1 cells to 115±2.1%, 126±3.6% and 119±3.3% of the untreated cells (*P*<0.05 *vs.* control; n = 48 in each group, Fig. 2*A*). The co-treatment with Akt inhibitor SH-6 (10 µM) or the AGE lowering reagent alagebrium (50 µM) prevented the MG-induced cell proliferation (Fig. 2*B*). When 3T3-L1 cells were treated with MG (5∼50 µM), the GSH level was significantly decreased. Consistent with the results from animal study, gloxalase I activity were not significantly altered by MG treatment (Fig. 2*C, D*).

**Figure 3:**
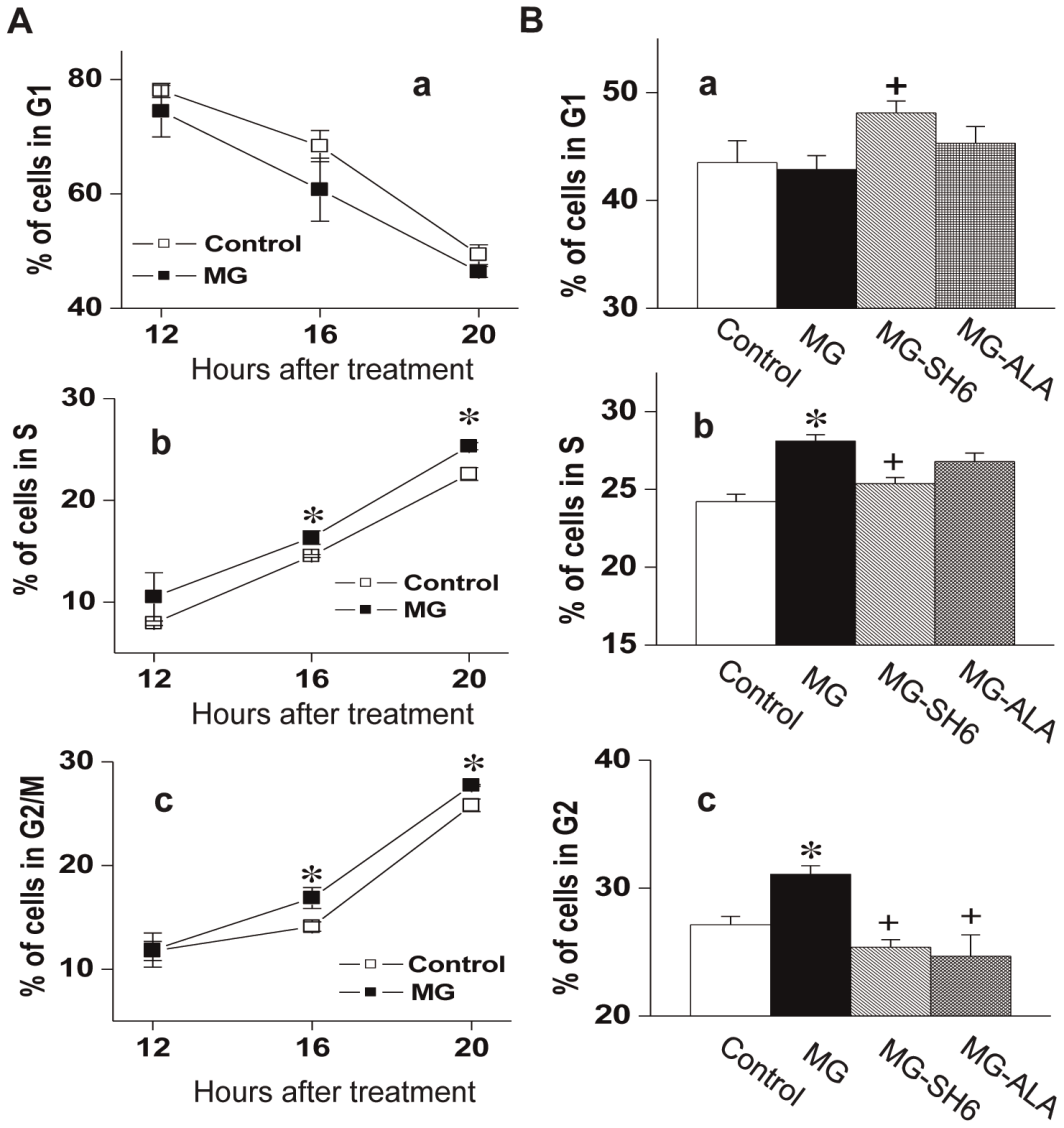
Effect of MG on cell cycle progression of 3T3-L1 cells. After 12, 16, 20 h of MG (10 µM) treatment, cellular DNA content was determined by a flow cytometer (***A***). The effect of MG with/without SH6 (10 µM) or alagebrium (50 µM) on cellular DNA content is shown in (***B***). *P<0.05 *vs* control group; **^+^** *P*<0.05 *vs* MG treated group; n = 6 in each group. The indicated percentage of the cell number is average of three experiments. CT: control; ALA: alagebrium.

The effect of MG on cell proliferation was further confirmed by analysis of the cell cycle phase distribution after MG treatment (Fig. 3). Comparing the cell number distributed in G_1_, S and G_2_ cell phase at different time points, we found that the MG-treatment lead to a faster cell cycle progression (Fig. 3*A*), which represented as an increased cell number in S phase after 16 or 20 h of MG (10 µM) treatment (Fig. 3*A* *–b*) and increased cell number in G_2_ phase after exposure of cells to MG (10 µM) for 20 h (Fig. 3*A* *–c*). The co-administration of SH-6 (10 µM) reversed the effect of MG on cell cycle progression in S and G_2_ phases (Fig. 3*B* *–b, c*). In contrast, increased G1 phase distribution indicated a longer interdivision time due to the inhibited rate of mass synthesis in cells co-treated with MG and SH-6, which reflects the inhibitive effect of SH-6 on Akt. Correspondingly, there was an increase of S phase cell number in cells treated with MG alone (Fig. 3*B* *–b*). Although reported to induce cell apoptosis at higher concentrations, 10 µM of MG did not increase sub-diploid/apoptotic cells. The percentage of apoptotic cells was 2.57%, 2.74%, 2.66% and 2.45% respectively for control group, MG-treated group, MG-SH6 and MG-ALA group.

### Effect of MG on Akt1 and its Downstream Targets in 3T3-L1 Cells

**Figure 4:**
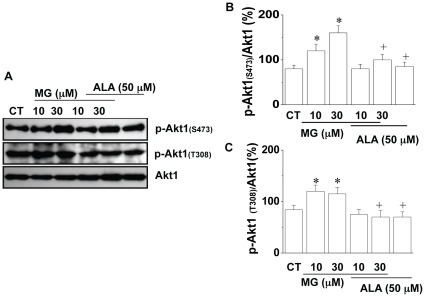
Effect of MG on Akt1 phosphorylation in 3T3-L1 cells. After 24 h treatment with or without MG (10 µM) in the presence or absence of SH-6 (10 µM)/alagebrium (50 µM), the protein levels of Akt1, (***A***), Representive Western blot of phospho-Akt1 (p-Akt1(Ser473), p-Akt1(thr308)) and Akt1; (***B***), The level of phospho-Akt1(Ser473) in 3T3-L1 cells with/without MG treatment; (***C ***), The level of phospho-Akt1(thr308) in 3T3-L1 cells with/without MG treatment. *P<0.05 *vs* control (CT) cells; **^+^** *P*<0.05 *vs* MG treated cells. The results were based on data from three experiments. CT: control; ALA: alagebrium.

To further understand the mechanism of MG induced cell proliferation in 3T3-L1 cells, the phosphorylation of Akt1 isoform was studied in cultured cells. Consistent with our observations of Akt1 in Zucker obese and lean animals, the levels of phospho-Akt1(Ser473) and phospho-Akt1(Thr308) in cultured 3T3-L1 cells were significantly increased after MG treatment (10 and 30 µM) for 24 h (Fig. 4). Co-application of alagebrium (50 µM) with MG attenuated the phosphorylation levels on both Ser473 and Thr308 of Akt1. Alagebrium itself had no significant effect on phospho-Akt1.

**Figure 5:**
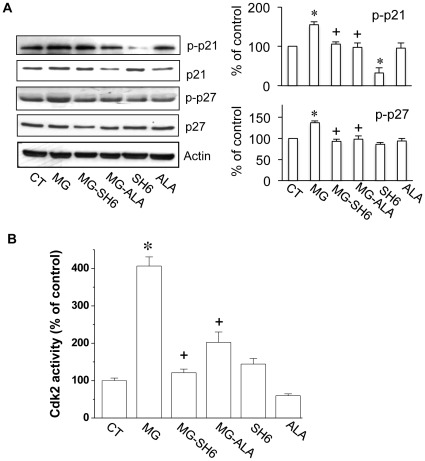
Effect of MG on p21, p-p21, p27, p-p27 and CDK2 activity in 3T3-L1 cells. After 24 h treatment with or without MG (10 µM) in the presence or absence of SH-6 (10 µM)/alagebrium (50 µM), the protein levels of p21, p-21 and p27, p-p27 (***A***), and the activity of Cdk2 (***B***) were determined and compared. *P<0.05 *vs* control (CT) cells; **^+^** *P*<0.05 *vs* MG treated cells. The results were based on data from three experiments.

As Akt regulates cell growth by phosphorylating p21 and p27 [26], we further examined the effect of MG on p21 and p27 in 3T3-L1 cells (Fig. 5). Consistent with the increased phosphorylation of Akt1, phosphorylated p21 (p-p21) and p27 (p-p27) were also observed in 10 µM MG treated 3T3-L1 cells (Fig. 5*A*), indicating a role of MG in stimulating Akt1 signaling. Co-administration of SH-6 (10 µM) or alagebrium (50 µM) significantly prevented the increased phosphorylation of p21 and p27 induced by MG.

In another group of experiments, we also examined the effect of MG on Cdk2 activity in 3T3-L1 cells. As shown in Fig. 5*B*, after the cells were treated with MG (10 µM) for 24 h, the activity of Cdk2 was increased to ∼4-fold of the control level. The increased Cdk2 activity was prevented by co-administration of either SH-6 or alagebrium. However, no significant change in the protein levels of Cdk2 in the cells treated with or without MG (10 µM) for 24 h was observed (data not shown).

### MG-treatment Resulted in More Lipid Accumulation in 3T3-L1 Cells

**Figure 6:**
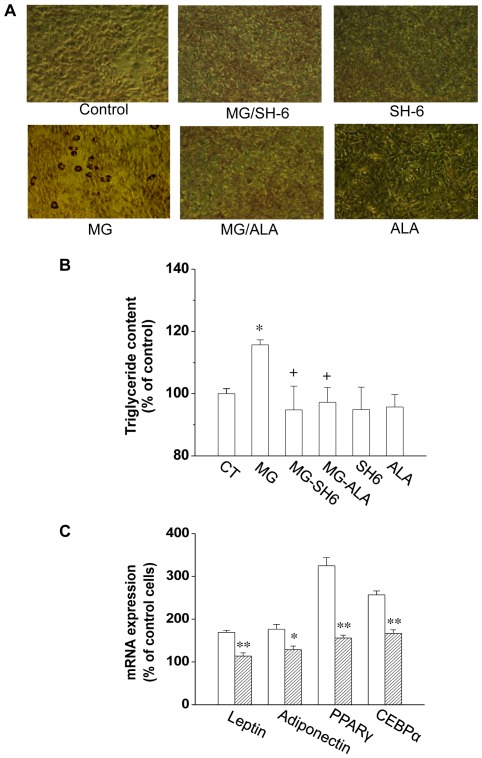
MG induced adipogenesis in 3T3-L1 adipocytes. After treated with MG, SH-6 or alagebrium for 48 h, cells were cultured till confluence and differentiation. The Oil Red O staining in adipocytes was shown in (***A***). The lipid content in adipocytes from different groups was quantified and presented as the percentage of that from control cells (***B***). The mRNA expression of adiponectin, PPARγ, C/EBPα and leptin in differentiated cells treated with MG alone or with MG and alagebrium were determined by real-time PCR (***C***). *P<0.05; **P<0.01; n = 3 in each groups. The open square in Figure 6*C* represents cells treated with MG; the stripped square represents cells treated with MG alagebrium. CT: control; ALA: alagebrium.

We have observed that incubation of 3T3-L1 cells with MG (10 µM) caused increased cell proliferation. To investigate whether this associates with a greater number of differentiated adipocytes, we treated the 3T3-L1 cells with MG, without or with SH-6 or alagebrium, for 48 h. On the 5th day of post-differentiation, triglyceride accumulation was indeed increased to 115.7±1.6% of the control level (Fig. 6*A, B*). The increased lipid content in MG-treated cells was attenuated by SH-6 or alagebrium co-administration. MG treatment (10–30 uM) of 3T3-L1 cells not only upregulates the transcriptional expression of adiponectin, leptin, PPARγ and C/EBPα, four important adipogenic markers, but also increases cellular adipogenesis (Fig. 6*C*). Furthermore, co-treatment of the cells with ALA efficiently reversed the MG-induced upregulation of these adipogenic markers.

## Discussion

Increased MG levels and MG-related AGEs have been reported in different insulin resistance states, which is associated with various clinical manifestations such as hypertension and diabetes [8], [11], [39]. However, the correlation between endogenous MG accumulation and the development of obesity, the major risk factor for insulin resistance, has not been shown previously. Our data in this study revealed the higher concentrations of MG in adipose tissue of obese Zucker rats. The increased basal level Akt1 phosphorylation observed in obese Zucker rats may represent the consequence of increased MG level in adipose tissue. However, it may also be related to the increased plasma insulin in obese rats which was observed in our previous study [40]. To further identify the effect of MG on Akt1 phosphorylation, we treated 3T3-L1 cells with MG. MG treatment induced the increases in the phosphorylation of Akt1, p21 and p27, as well as an enhanced activity of Cdk2 and accelerated cell cycle progression. This result reinforces the correlation between MG accumulation and Akt1 phosphorylation observed in animal model. Thus, increased MG may be one of the factors that contribute to increase Akt phosphorylation in obese animals.

The deleterious effect of MG on different types of cells has been extensively studied [30], [31], [32]. A previous study reported an increased apoptotic cell number when mouse Schwann cells were treated with 500–1000 µM MG [31]. In another study, MG (100 µM, [30] altered the PDGF-induced PDGFR_β_-phosphorylation, and reduced the proliferation of mesenchymal cells (smooth muscle cells and skin fibroblasts). The major difference in our study is that the 3T3-L1 cells were treated with more physiologically relevant concentrations (1.25∼20 µM) of MG compared with 0.2∼5 µM of MG in normal human/rats based on previous and the present studies [37], [41]. In agreement with previous studies, we observed a decreased proliferation of 3T3-L1 cells when the MG concentration was increased to 100 µM (data not shown). This might indicate a biphasic effect of MG on cell proliferation. Most probably, the inhibitory effect of MG is due to the acute effect of high MG concentration, but not the effect of MG at the physiological relevant level. To our knowledge, this is the first report about the stimulating effect of MG on pre-adipocytes proliferation. However, this effect may not be limited to adipocytes. Increased proliferation was also observed in vascular smooth muscle cells after MG treatment [42] in our other study. In response to increased MG level, 3T3-L1 cells showed a decreased GSH level, but glyoxalase I activity remained unchanged (Fig. 2*C, D*). Obese rats showed a similar pattern of change in GSH level and glyoxalase I activity as observed in MG treated cells. The unchanged glyoxalase I activity suggests that increased MG accumulation in obese rats may mainly due to increased MG production. As the consumption of high-carbohydrate food has increased worldwide, the prevalence of metabolic syndrome such as diabetes and obesity has also risen. The usage of table sugar sucrose (1 glucose +1 fructose) and high fructose corn syrup (HFCS, 55% fructose and 45% glucose) to sweeten beverages (such as soft drinks) or as an ingredient in processed foods has increased significantly in the last several decades. Obese Zucker rats lack leptin receptor. Leptin exerts a negative control on food intake. Increased food intake due to the absence of leptin receptor provides a surplus of substrate for MG production in these obese Zucker rats. Elevated vascular tissue MG levels were reported in normal SD rats fed with high fructose (60% in diet) for 16 weeks and in cultured vascular smooth muscle cells treated with high fructose (25 mM). Putative mechanisms for this MG overproduction are increased cellular fructose accumulation due to the upregulation of Glut5 (a transporter for fructose) and aldolase B (a key enzyme that catalyzes MG formation from fructose) [43]. In obese Zucker rats, MG levels are elevated in plasma, adipose tissues, and kidney, but not in the liver (*Fig 1*). The mechanisms for MG overproduction in different tissues or organs might be different and whether adipose tissue and vascular tissue share the similar mechanism needs further investigation.

The possible underlying mechanism for MG effects on cell proliferation may related to dAkt1 signal cascade, which plays an important role in regulating cell proliferation. Based on our results, the effect of MG on cell proliferation was due to MG-increased activity of Akt1 and the related targets. In our study, 10 µM of MG in cultured 3T3-L1 cells increased the phosphorylation of Akt1. Furthermore, MG treatment increased the phosphorylation of p21 and p27 (Fig. 5*A*), the major regulators that arrest the cell cycle progression at the G_1_/S checkpoint. The increased phosphorylation of p21 and p27 activates their degradation and leads to the entry of cells to S phase. This explains the MG-activated cell proliferation detected in our experiment. Further observation of the increased Cdk2 activity in MG-treated cells supported this hypothesis (Fig. 5*B*). Increased Akt1 activity was observed in MG-treated 3T3-L1 cells suggesting that change in Akt1, especially its activity, is critical in adipocyte proliferation. The MG-treated cells also showed more lipid accumulation as well as increased expression of adipogenic markers. It may be a direct result of increased cell numbers after MG was administered during the proliferation stage (Fig. 6).

In the current study, Akt1 not only shows increased phosphorylation level in MG treated adipocytes, but also in adipose tissues from obese rats with an increase of MG accumulation (Fig. 1, 4 and 5). Moreover, the increased phosphorylation on Akt1 and its targets (p21 and p27) was efficiently attenuated by AGE lowering reagent alagebrium. According to our previous study, modification of a cysteine residue in Akt1 may be favorable for the activation of Akt1 by phosphorylation on activation sites Ser (473) and Thr (308) [42]. Findings in this paper suggest that MG mediated Akt1 modification and activation may also contribute to the increased adipocyte numbers and enlargement of adipose tissue. Alagebrium is an AGE lowering agent. Previous studies have reported its role in attenuating the accumulation of extracellular matrix in humans and animals with diabetic complications. In this study, alagebrium inhibited the proliferation-provoking effect of MG. It is unlikely that alagebrium impeded Akt1 activity directly. Instead, it may function by reversing the MG-induced modification of selective amino acid residues of Akt1 protein. Various doses (1–100 µM) of alagebrium have been tested in our preliminary studies. We found that 50 µM was the optimized concentration of alagebrium and therefore this concentration was applied throughout the entire work. This concentration seems higher than the previous reported doses (1–10 µM) to prevent extracellular matrix and neointima formation [44]. The dosage of alagebrium applied in different studies might also relate to different cell types and experimental conditions. We observed an inhibited Cdk2 activity after ALA treatment. The most possible reason might still due to the AGE-lowering effect of ALA because ALA would scavenge the endogenous produced MG from the cultured cells. However, the underlying mechanisms of this inhibitive effect need further investigation.

Obesity in childhood involves both adipocyte hyperplasia and hypertrophy while adult-onset obesity was generally considered due to adipocyte hypertrophy. However, various lines of evidence indicate that adipocyte hyperplasia is also an important factor in the development of adult-onset obesity, especially morbidly obese patients with BMI value >39. Since the food intake of obese Zucker rats is more than lean rats and food over-consumption can lead to increased MG formation, it is hard to evaluate the extent of contribution from MG to the development of obesity in Zucker rats used in this study. However, the results in this study suggest that MG-stimulated adipogenesis by the up-regulation of Akt signaling pathway may be a new explanation to the development of obesity especially the adult-onset morbid obesity.

## Materials and Methods

### Animals

Eight 8-week-old male obese Zucker rats and eight age-matched lean Zucker rats were purchased from Charles River laboratories, Inc. (Wilmington, MA), housed in temperature-regulated animal facility and maintained at 22–23°C. These animals were exposed to a 12 h light/dark cycle with free access to water and food. The standard lab rat chow, Prolab® RMH 3000, contains 60% starch, 22% crude proteins, 5% crude fat, 5% crude fiber, 6% ash, and 2% added minerals (PMI® Nutrition International, St. Louis, MO). Rats were treated in accordance with guidelines of the Canadian Council on Animal Care and the experimental protocols were approved by the Animal Care Committee of the University of Saskatchewan. At the end of week 16, after overnight fasting, rats will be anaesthetized with sodium pentobarbital (50 mg/kg body weight) injected intraperitoneally. Kidney, fat and liver tissues were collected and frozen under −80°C.

### Culture and Differentiation of 3T3-L1 Cells

3T3-L1 pre-adipocytes were grown to confluence in Dulbecco’s modified Eagle’s medium (DMEM, Invitrogen, ON, Canada) containing 10% bovine calf serum (Invitrogen, ON, Canada). At two days postconfluence, cell differentiation was induced by adding insulin (2.5 µg/ml, Sigma, St Louis, MI, USA), dexamethasone (0.25 µM, Sigma-Aldrich, MO, USA), and isobutylethylxanthine (IBMX, 0.5 mM, Sigma-Aldrich, MO, USA) to media for 3 days according to the protocol described previously [45]. The cells then were grown in post-differentiation media (DMEM containing 10% fetal calf serum and 2.5 µg/ml insulin). The post-differentiation medium containing different concentrations of MG and/or AGE lowering reagent alagebrium was changed every day until cells were differentiated. Cells were collected by trypsin digestion after treatments.

### MG Measurement

Quantitation of MG used the o-phenylenediamine (o-PD)-based assay as described by Chaplen *et al* [46], with some modifications [11]. Briefly, the supernatant of tissue homogenate or serum was incubated with 100 mmol/L o-PD for 3 h at room temperature. The quinoxaline derivative of MG (2-methylquinoxaline) and the quinoxaline internal standard (5-methylquinoxaline) were then measured using a Hitachi D-7000 high-performance liquid chromatography (HPLC) system (Hitachi Ltd., Mississauga, Ontario, Canada).

### Western Blotting

The supernatants containing crude cellular proteins were resolved on a 12% SDS-PAGE gel, and transferred onto the PVDF membrane (PALL Corporation, Ontario, Canada). The membrane was blocked and incubated with different primary antibodies overnight. After washed 3 times with the PBS-T for 30 min, the membrane was incubated with the HRP-conjugated secondary antibody for 1 h at room temperature. The immunoreactions were visualized by ECL and exposed to X-ray film (Kodak Scientific Imaging film, X-omat Blue XB-1). The Western bands were then quantified using Chemigenus® Bio imaging system and normalized by β-actin.

### Cell Proliferation Assay

The proliferation of 3T3-L1 cells was measured by the Celltiter 96® non-radioactive cell proliferation assay kit (Promega, WI, USA). Briefly, cells were seeded onto 96-well plates (5000 cells per well) and cultured in Dulbeco’s Modified Eagle’s Medium (DMEM, HyClone, Ontario, Canada). When they reached ∼50% of confluence in medium, the medium was removed and the cells were washed with serum-free medium and incubated in serum-free medium for 48 h. The cells were then treated with/without MG, SH-6 (10 µM, *Calbiochem,* California, USA) or alagebrium (50 µM, gift from Synvista Therapeutics, Inc. NJ, USA) for 48 h in serum-containing DMEM medium supplemented. The 40% methylglyoxal solution was from Sigma-Aldrich (MO, USA). To remove the impurities, this commercial solution was further purified by fractional vacuum distillation. The final concentration of MG was determined by HPLC and used in the present study. After treatment, the cells were incubated with dye solution (15 µL for each well) in medium at 37°C for 4 h and then incubated with solubilization solution at room temperature for 1 h. The spectrophotometric absorbance of the samples was determined by using a plate reader (Thermo Labsystems, Finland) at 570 nm.

### Cell Cycle Assay

Cell cycle analysis was performed by propidium iodide (PI) staining. Briefly, 3T3-L1 cells were firstly seeded into 10 cm dishes. When they reached ∼50% of confluence, the cells were incubated in serum-free medium for 48 h and then treated with MG, SH-6 (10 µM) or alagebrium (100 µM) for 12, 16 or 20 h. Subsequently, the cells were harvested and re-suspended in PBS at 1×10^6^/mL and fixed with 70% cool ethanol for 1 h. After the cells were washed and centrifuged, the pelleted cells were re-suspended in 1 mL PBS and added with 50 mL of RNase A stock solution (10 g/mL). Followed a 3 h incubation at 4°C, the cells were then pelleted and added with 1 mL of PI staining solution (3.8 mmol/L sodium citrate, 50 mg/mL PI in PBS) and analyzed by flow cytometry on an Beckman Coulter Epics XL flow cytometer (Beckman Coulter Canada Inc, Ontario, Canada). The number of cells counted in each run was 1∼2×10^5^ cells.

### Measurement of Glutathione (GSH) Level and Glyoxalase I Activity

The monochlorobimane procedure was used to measure GSH contents as described previously [47]. The GSH-monochlorobimane adduct was measured using a Thermo Labsystems, Finland microtitre fluorometric reader with excitation at 380 nm and emission measured at 470 nm. The activity of glyoxalase I was evaluated by monitoring the increase in absorbance at 240 nm due to the formation of S-D-lactoylgutathione in the presence of homogenates. Protein concentrations were determined by bicinchoninic acid procedure using bovine serum albumin as the reference.

### CDK2 Activity Assay

CDK2 activity was determined by measuring ATP consumption with PKLight Assay Kit (Cambrex Bio Science, ME, US) as described before [42]. Briefly, after incubation of 200 µg of proteins with 2 µg of anti-CDK2 antibody (Santa Cruz) in cell lysis buffer for 4 h at 4°C, protein A/G plus agarose beads (20 µL) were added and the mixture was incubated overnight at 4°C with shaking. Beads were washed 3 times and suspended in 40 µL of CDK2 kinase assay buffer containing 20 µM ATP and 0.1 µg/µL histone H1. Above mixture was reacted at 30°C for 30 min in 96-well plate before kinase stop solution and ATP detection reagent were added according to the manufacture’s protocol. Bioluminescent signal in each well was detected using a microplate spectrofluorometer (BMG LABTECH Inc., NC, US). CDK2 activity was expressed as ATP consumption from 3 experiments.

### Adipogenesis Assay

3T3-L1 cells were treated with/without MG, SH-6 (10 µM or alagebrium (50 µM) for 48 h and then continue cultured in fresh medium. When the cells reached 80% confluence, differentiation was induced by adding 2.5 µg/ml insulin, 0.25 µM dexamethasone (Sigma-Aldrich, MO, USA), and 0.5 mM isobutylmethylxanthine (Sigma-Aldrich, MO, USA) to media for 2 days according to the protocol described previously [45], [48]. The cells were then grown in post-differentiation medium (DMEM containing 10% fetal calf serum and 2.5 µg/ml insulin). On the fifth day of post-differentiation, cells were fixed with 4% (v/v) formaldehyde in PBS, and then stained with Oil Red solution for 15 min. The cells were rinsed five times with water and pictures were photographed under microscope. Cells were considered as being lipid-positive when droplets were stained red. The dye retained by the cells was eluted by incubation with 500 µl of isopropanol and quantified by measuring absorbance at 500 nm by a plate reader (Thermo Labsystems, Finland).

### Real-time PCR

For total-RNA preparation, treated 3T3-L1 cells were homogenized in TRIzol® reagent (Invitrogen, ON, Canada) and RNA was isolated according to the manufacturer’s instructions. Total RNA was reverse-transcribed in triplicate using RevertAid™ H Minus M-MuLV reverse transcriptase (MBI, Fermentas Burlington, ON, Canada) in the presence of 5×RT buffer (MBI, Fermentas, MD, USA), random primer (Invitrogen, Burlington, ON, Canada), dNTP mixture (Amersham Pittsburgh, PA, USA) at 42°C for 50 min, followed by 72°C for 10 min. The primers of Leptin were 5′- CGGGGCGCTGGTGTAGGGAGATT -3′ (sense) and 5′- ACACCGCATGGAGAGTCGCAGGAG -3′ (antisense). The primers of adiponectin were 5′-ATGGGTAGTTGCAGTCAGTTGGTA -3′ (sense) and 5′- GCCGCTTATGTGTATCGCTCAG -3′ (antisense), The primers of PPARγ were 5′- AGGGCTTCCGCAGGTTTTTGA -3′ (sense) and 5′- CACAGGCCGAGAAGGAGAAGC -3′ (antisense). The primers of C/EBPα were 5′- CTTGCGCAGGCGGTCATTGTCACT -3′ (sense) and 5′- GGCCGGCCTCTTCCCCTACCAG -3′ (antisense), The real-time PCR was carried out in an iCycler iQ apparatus (Bio-Rad, CA, USA) associated with the ICYCLER OPTICAL SYSTEM software (version 3.1) using SYBR Green PCR Master Mix (Bio-Rad).

### Data Analysis

Data are expressed as mean ± SEM and analyzed using one way ANOVA in conjunction with *t* test where applicable. Significant difference between treatments was defined at a level of *P*<0.05.
